# Protective Effect of Spin-Labeled 1-Ethyl-1-nitrosourea against Oxidative Stress in Liver Induced by Antitumor Drugs and Radiation

**DOI:** 10.1155/2013/924870

**Published:** 2013-09-24

**Authors:** V. Gadjeva, B. Grigorov, G. Nikolova, A. Tolekova, A. Zheleva, M. Vasileva

**Affiliations:** ^1^Department of Chemistry and Biochemistry, Faculty of Medicine, Trakia University, Armeiska Street 11, 6000 Stara Zagora, Bulgaria; ^2^Department of Physiology, Pathophysiology and Pharmacology, Faculty of Medicine, Trakia University, Armeiska Street 11, 6000 Stara Zagora, Bulgaria; ^3^Laboratory of Oncopharmacology, National Cancer Institute, 1756 Sofia, Bulgaria

## Abstract

This study was carried out to investigate possible protection effect of 1-ethyl-3-[4-(2,2,6,6-tetramethylpiperidine-1-oxyl)]-1-nitrosourea (SLENU), synthesized in our laboratory, against oxidative liver injuries induced in mice treated by antitumor drugs: doxorubicin (DOX), bleomycin (BLM), or gamma irradiation (R). Specifically, alterations in some biomarkers of oxidative stress, such as lipid peroxidation products measured as malondialdehyde (MDA) levels and activities of the antioxidant enzymes, superoxide dismutase (SOD) and catalase (CAT), were studied in liver homogenates isolated from tumor bearing C57 black mice after i.p. treatment with solutions of DOX (60 mg/kg), BLM (60 mg/kg), or after total body gamma-irradiation with a single dose of 5 Gy. The same biomarkers were also measured after i.p. pretreatment of mice with SLENU (100 mg/kg). Statistical significant increased MDA levels and SOD and CAT enzymes activities were found in the liver homogenates of tumor bearing mice after alone treatment with DOX or gamma-irradiation compared to the control mice, while these parameters were insignificantly increased after BLM administration compared to the same controls.

## 1. Introduction 

Modern chemotherapy, along with surgery and radiation therapy, is still the most efficient method of cancer treatment. The final common pathway in the mechanisms of action of ionizing radiation and many chemotherapeutic agents include alterations of DNA and the production of reactive oxygen species (ROS) [1, 2]. In particular, double-strand breaks have a major impact on cell killing after irradiation. The increased production of ROS, however, could be a reason for many dangerous side effects that sometimes hamper the therapy and may lead to serious or even fatal organ dysfunctions.

Among the anticancer drugs, doxorubicin and bleomycin have been used for the treatment of many malignant tumors. Although these drugs belong to different classes, doxorubicin is an anthracycline glycoside antibiotic, whereas bleomycin is a glycosylated peptide antibiotic, they share some properties. Thus, ROS were shown to be involved in the toxicity of both doxorubicin and bleomycin [3, 4]. Also, chronic organ toxicity frequently develops upon administration of cumulative doses of both drugs. Finally, interactions of both drugs with iron are considered to be of importance in exerting their deleterious effects on healthy tissues as well as in their antineoplastic activity [5, 6]. Bleomycin has been used for the treatment of germ cell tumors, lymphomas, Kaposi's sarcomas, and so forth. Bleomycin is considered radiomimetic and oxidative DNA-cleaving reagent [7]. The clinical usefulness of BLM is restricted, since it has several acute and chronic side effects. The most serious complications of BLM are pulmonary fibrosis and impaired lung function. Minor important adverse effects are myelosuppression, nauseas, vomiting, allergic reactions, mucositis, alopecia, erythema, hyperkeratosis, hypopigmentation, skin ulceration, and acute arthritis [8]. Hepatotoxicity is also minor and reversible [4, 9]. Doxorubicin possesses a potent and broad-spectrum antitumor activity against a variety of human solid tumors and hematological malignancies. However, its use in chemotherapy has been limited largely due to its diverse toxicities. Reactive oxygen species, generated by the interaction of doxorubicin with iron, can damage cellular systems, with the most serious adverse effect being life-threatening heart damage. Other tissues, like the kidneys, brain, liver, and the skeletal muscles, are also affected by DOX [10, 11]. Chemotherapy with DOX can cause liver abnormalities such as ascites, hyperbilirubinemia, reactivation of hepatitis B, and thrombocytopenia leading to fatalities [12–14].

At least 50 percent of all cancer patients receive radiotherapy at some stage during the course of their illness. Radiotherapy is currently used to treat localized solid tumors, such as cancers of the skin, brain, breast, or cervix, and can also be used to treat leukemia and lymphoma [15, 16]. However, a number of patients undergoing radiation therapy experience a range of side effects, which may lead to an interruption of treatment or limiting the dose of radiation. A growing body of evidence appears to support the hypothesis that oxidative stress might serve to drive the progression of radiation-induced toxic side effects [17–19]. Free radicals are considered to be the common mediator of DNA damage after ionizing radiation. Radiation's effects on normal tissues occur predominantly in slowly growing tissues such as the lungs, liver, kidneys, heart, and central nervous system [15]. 

Strategies to attenuate drugs and radiation toxicity include dosage optimization, synthesis, and the use of analogues having lower toxicity or a combined therapy with antioxidants. Clinical and experimental trials have been directed toward employing various antioxidant agents to ameliorate drug- and radiation-induced liver damage. The most promising results come from the combination of the drug delivery together with an antioxidant in order to reduce oxidative stress. Although a number of studies have examined the protective effects of antioxidants such as vitamins C and E, carotenoids, and selenium, these studies have not provided consistent evidence in favor of hepatotoxic effects of the anthracyclines and radiation [20]. Other compounds such as erdosteine, cystathionine, and catechin might also prevent oxidative liver injury induced by these antitumor drugs and radiation [21, 22].

Stable nitroxyl radicals such as 4-hydroxy-2,2,6,6-tetramethylpiperidine-*N*-oxyl (Tempol) have been shown to function as superoxide dismutase (SOD) mimics and to protect mammalian cells against superoxide and hydrogen peroxide-mediated oxidative stress and radiation-induced cytotoxicity [23]. Reduced toxicity and increased antineoplastic properties were achieved when nitroxyl (aminoxyl) groups were introduced in chemical structure of certain antitumor drugs [24, 25]. This finding encourages us to synthesize a number of spin-labeled analogues of the anticancer drug 1-(2-chloroethyl)-3-cyclohexyl-1-nitrosourea (CCNU). Some of these compounds showed advantages over CCNU, having lower toxicity and higher anticancer activity against some experimental tumor models [26, 27]. By EPR method, we have shown that spin-labeled nitrosoureas and their precursor 4-amino TMPO can scavenge ^•^O_2_^−^ and so exhibit high superoxide-scavenging activity (SSA) [28]. Moreover, by our studies, we have demonstrated beneficial effects of SLENU, recently synthesized in our laboratory, analogue of the antitumor drug CCNU, and vitamin E as positive control on CCNU-free radical-induced oxidative injuries in rat blood and in liver of mice [29, 30].

**Figure 1:**
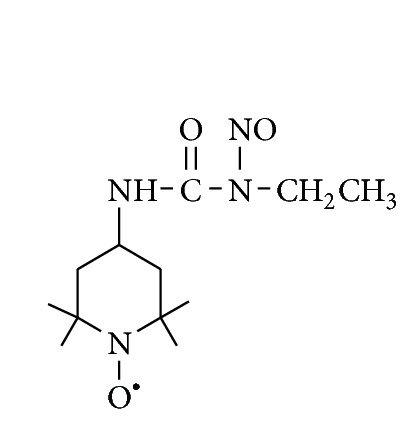
Chemical structure of 1-ethyl-3-[4-(2,2,6,6-tetramethylpiperidine-1-oxyl)]-1-nitrosourea (SLENU).

Therefore, the aim of the present study was to investigate whether, pretreatment with spin-labeled nitrosourea SLENU (Figure 1) possessing high SSA would decrease liver oxidative stress injuries in mice induced by application of antitumor drugs or gamma irradiation. To achieve the ultimate goal of this research, we investigated the levels of lipid peroxidation and activities of antioxidant defense enzymes superoxide dismutase (SOD) and catalase (CAT) in liver homogenates of tumor bearing mice treated by the antitumor drugs doxorubicin, bleomycin, or after total body irradiation alone and compared to the levels of the same parameters measured after pretreatment with SLENU.

## 2. Materials and Methods

### 2.1. Drugs and Chemicals

Bleomycin and Farmorubicin were obtained from Bristol Myers Squibb, Wallingford, CT, USA. Buttermilk xanthine oxidase, trolox, SULF (sulfanilamide), NEDD (N-(1-naphthyl) ethylenediamine dihydrochloride), and VCl_3_ were obtained from Fluka (Germany). TMPO was purchased from Aldrich (Milwaukee, USA). SLENU was synthesized according to Gadjeva and Koldamova [31]. The test compounds were dissolved ex tempore: first step in Tween and second step in saline. 

### 2.2. Experimental Animals

All procedures performed on animals were done in accordance with guidelines of the Bulgarian government regulations and were approved by the authorities of Trakia University. The animals were housed in plastic cages, fed a normal laboratory diet and water ad libitum. 

The study was carried out on 142 C57 black mice (bred in the Laboratory of Oncopharmacology, National Cancer Institute, Sofia), with average weight of 18–22 g, divided into groups of 6 animals per group (equal number of the two sexes). 

### 2.3. Experimental Design

The blood for the analysis was taken by a heart puncture after opening the thoracic region. The venous blood samples were divided into portions. The serums were used for an analysis of enzymatic activities and the level of NO. Mice were sacrificed by cervical decapitation at 1 hour after administration of the drugs or gamma irradiation. Livers were removed and kept on ice until homogenization on the same day. The samples were first washed with deionized water to separate blood and then homogenized. The tissue homogenates were centrifuged at 15 000 rpm for 10 minutes, 4°C and the final supernatants were obtained. They were used for determination of lipid peroxidation and the activities of superoxide dismutase and catalase.

#### 2.3.1. Drug Treatment

On day 0, mice were inoculated i.p. with 10^5^ tumor cell suspension from lymphoid leukemia L1210 in saline in volume of 0.5 mL. On day 3, Bleomycin (60 mg/kg), Farmorubicin (60 mg/kg), in accordance with LD50 of the drugs, spin-labeled nitrosourea SLENU (100 mg/kg), and combinations of them were administrated i.p. in a single injection in volume 0.01 mL per body weight, as 10% Tween solutions in accordance with the routine methods described in the literature [32]. 

#### 2.3.2. Irradiation

Total-body irradiation of mice was performed with an orthovoltage Philips RT-250 irradiator, 225 kVp X-ray source, operating at 15 mA and filtered with 0.2 cm copper. Mice were exposed to 5 Gy total-body *γ*-irradiation at a dose rate of 2,52 cGy/s in the absence or presence of injected SLENU (100 mg/kg), 10 min after administration. All animals were weighed prior to irradiation. After irradiation animals were returned to the animal facility.

### 2.4. Investigation of Oxidative Stress Parameters

#### 2.4.1. Analysis of Lipid Peroxidation in Liver

Basal levels of lipid peroxidation as indicated by thiobarbituric acid-reactive substances (TBARS) were determined using the thiobarbituric acid (TBA) method, which measures the malondialdehyde (MDA) reactive products [33]. In the TBARS assay, 1 mL of the supernatant, 1 mL of normal saline, and 1 mL of 25% trichloroacetic acid (TCA) were mixed and centrifuged at 2000 rpm for 20 minutes. One mL of protein-free supernatant was taken, mixed with 0.25 mL of 1% TBA and boiled 1 h at 95°C. After cooling, the absorbance of the pink color of the obtained fraction product was read at 532 nm. 

#### 2.4.2. Measurement of Antioxidant Enzymes Activities in Liver

Total SOD activity was determined by the xanthine/xanthine-oxidase/nitroblue tetrazolium (NBT) method according to Sun et al. [34], with minor modification. Superoxide anion radical (O_2_^−^) produced by xanthine/xanthine-oxidase system reduces NBT to formazan, which can be assessed spectrophotometrically at 560 nm. SOD competes with NBT for the dismutation of ^•^O_2_^−^ and inhibits its reduction. The level of this reduction is used as a measure of SOD activity. The total SOD activity is expressed in units/mg of protein, where one unit was equal to SOD activity that causes 50% inhibition of the reaction rate without SOD.

The assay of CAT activity was according to Beers Jr. and Sizer [35]. Briefly, hydrogen peroxide (30 mM) was used as a substrate and the decrease in H_2_O_2_ concentration at 22°C in a phosphate buffer (50 mM, pH 7.0) was followed spectroscopically at 240 nm for 1 min. The activity of the enzyme was expressed in units per mg of protein, and 1 unit was equal to the amount of an enzyme that degrades 1 *μ*M H_2_O_2_ per minute. 

#### 2.4.3. Measurement of NO^•^ in Serum

Serum nitric oxide was measured in terms of its products, nitrite and nitrate, by the method of Griess, modified by Miranda et al. [36]. This method is based on a two-step process. The first step is the conversion of nitrate to nitrite using vanadium (III) and the second is the addition of sulphanilamide and N (-naphthyl) ethylenediamine (Griess reagent). This converts nitrite into a deep-purple azo compound, which was measured colorimetrically at 540 nm. Nitric oxide products were expressed as *μ*M. 

### 2.5. Estimation of Serum Transaminases sGPT and sGOT

The liver function was evaluated with serum levels of glutamate oxaloacetate transaminase sGOT and glutamate pyruvate transaminase sGPT. The determination of sGOT and sGPT was based on the fact that phenylhydrazone, which produced after incubation the substrate with the enzyme, was measured spectrophotometrically [37]. The amount of phenyl hydrazone formed was directly proportional to the enzyme quantity.

### 2.6. Statistical Analysis

The data are expressed as a mean ± SE. Student's *t*-test was used to determine the statistical differences between groups. *P* < 0.05 was considered statistically significant.

## 3. Results

### 3.1. Effect of SLENU on sGPT and sGOT Levels

In the present study, the liver function was evaluated with serum levels of glutamate oxaloacetate transaminase sGOT and glutamate pyruvate transaminase sGPT, which were measured in the serum as markers of cellular injury. There were not significant changes of the levels of sGPT and sGOT between healthy and tumor bearing control mice. The levels of sGPT and sGOT were increased but not significantly (*P* > 0.05) after i.p. administration of a single dose of BLM (60 mg/kg) in tumor bearing mice. However, there was dramatic increase in the enzymes levels of sGPT and sGOT after i.p. administration of DOX in dose (60 mg/kg) and gamma irradiation with a single dose of 5 Gy in tumor bearing mice, compared to the untreated cancer control groups (*P* < 0.00001). When mice were pretreated with SLENU i.p. dose (100 mg/kg), 30 min. prior to DOX and gamma-irradiation, a statistical significant reduction was found in the levels of sGPT and sGOT compared to the groups treated with DOX or gamma irradiation alone (*P* < 0.0001) (Table 1).

### 3.2. Effect of SLENU on MDA Level and Antioxidant Enzymes SOD and CAT in Liver

**Figure 2:**
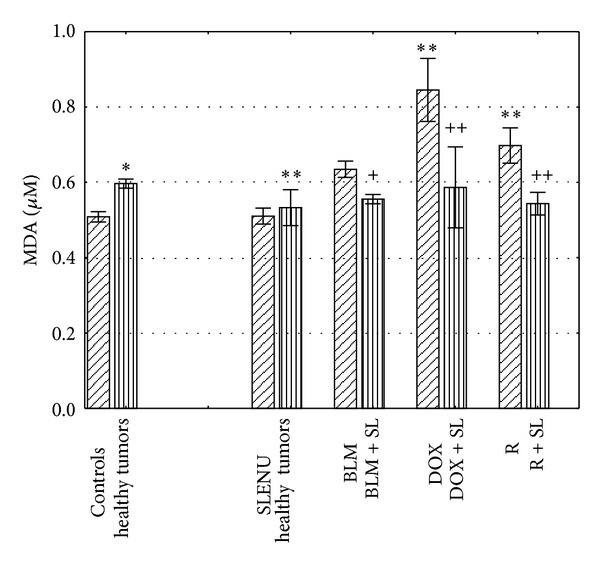
Lipid peroxidation in liver homogenates isolated from mice 1 hour after i.p. administration of BLM, DOX, and R alone and in combination with SLENU. Values are expressed as mean ± SE. *P < 0.001 versus health controls; **P < 0.0001 versus tumor controls ^+^ *P* < 0.01 versus group with BLM administrated alone; ^++^ *P* < 0.0001 versus group with DOX and R administrated alone.

The levels of lipid peroxidation in liver homogenates isolated from mice treated with BLM, DOX, and R alone and in combination with SLENU are shown in Figure 2. It was found that the levels of MDA were significantly increased in tumor bearing mice compared to the healthy controls (0.606 *μ*M versus 0.508 *μ*M, *P* < 0.001). No significant difference, compared to the healthy controls, was observed in the groups of either tumor bearing or healthy mice treated with SLENU (mean 0.488 U/gPr and 0.426 U/gPr, *P* > 0.05). One hour after administration of BLM, DOX and R, the levels of MDA were significantly increased in liver homogenates isolated from tumor bearing mice treated with DOX, and R, compared to the group of tumor controls (mean 0.893 *μ*M and 0.698 *μ*M, *P* < 0.0001) and not significantly increased in liver homogenates isolated from tumor bearing mice treated with BLM (mean 0.625 *μ*M, *P* > 0.05). Combined application of BLM and SLENU led to a decrease in the level of MDA compared to the level when BLM was administrated alone (mean 0.561 *μ*M, *P* < 0.01). However, combinations of either DOX or R with SLENU led to a strong decrease in the levels of MDA, compared to the levels when DOX and R were administrated alone (mean 0.569 *μ*M and 0.543 *μ*M, *P* < 0.0001); the levels of the former were close to those obtained from SLENU when administered alone. 

**Figure 3:**
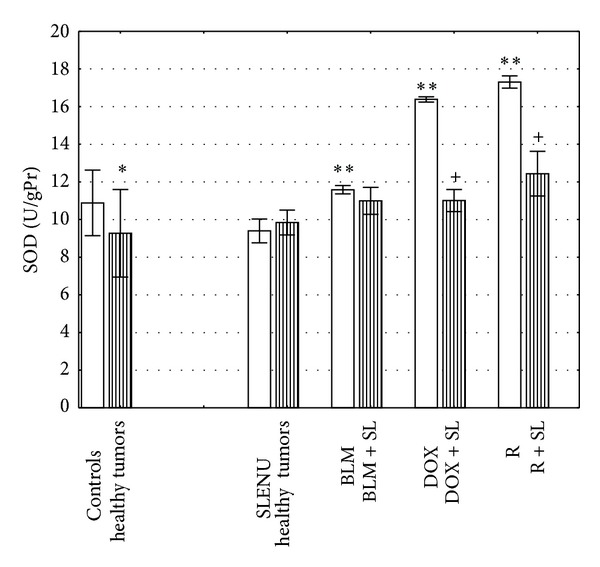
SOD activity of liver homogenates isolated from mice at 1 hour after administration of BLM, DOX, and R alone or in combination with SLENU. Values are expressed as mean ± SE. *P < 0.001 versus health controls; **P < 0.0001 versus tumor controls; ^+^ *P* < 0.05 versus group treated with DOX alone; ^+^ *P* < 0.0001 versus group with DOX and R administrated alone.

As can be seen from the data represented in Figure 3, the activities of SOD in liver homogenates isolated from tumor bearing control mice at 1 h were significantly decreased compared to SOD activities of liver homogenates, isolated from healthy controls (mean 8.472 U/gPr versus 10.882 U/gPr, *P* < 0.001). No significant difference, compared to the healthy controls, was observed in the groups of either tumor bearing or healthy mice treated with SLENU (mean 9.826 U/gPr and 8.688 U/gPr, *P* > 0.05). After treatment with BLM, DOX, and R alone, SOD activities of liver homogenates from tumor bearing were found to be significantly higher than those of the tumor bearing controls (mean 11.583 U/gPr, 16.213 U/gPr, and 17.306 U/gPr, *P* < 0.0001, resp.). However, a combined application of BLM, DOX, and R with SLENU had lower SOD activities for tumor bearing mice compared to the groups of tumor bearing mice treated with BLM, DOX, and R alone and was close to the healthy controls. A combined application of BLM and SLENU led to a decrease but not significant in the level of SOD compared to that of BLM administrated alone in tumor bearing mice (mean 10.924 U/gPr, *P* > 0.05). Moreover, in tumor bearing mice after administration of the combination of either DOX or R with SLENU, SOD activities were significantly decreased compared to those of DOX and R administrated alone (mean 10.698 U/gPr and 12.251 U/gPr, *P* < 0.0001) and were close to those of the healthy controls. 

**Figure 4:**
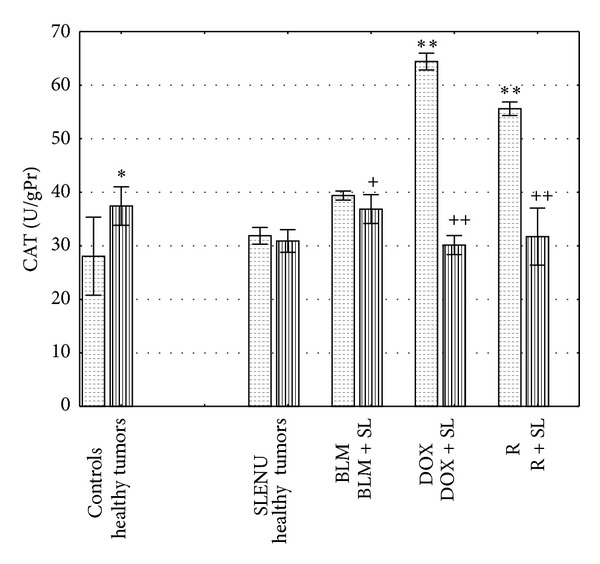
CAT activity of liver homogenates isolated from mice 1 hour after administration of BLM, DOX, or R alone or in combination with SLENU. Values are expressed as mean ± SE. *P < 0.0001 versus health controls; **P < 0.0001 versus tumor controls; ^+^ *P* < 0.001 versus group treated with BLM alone; ^++^ *P* < 0.00001 versus groups treated with DOX and R alone.

Figure 4 represents the activity of the antioxidant enzyme CAT in liver homogenates isolated from healthy and tumor bearing mice. The activity of CAT in tumor bearing control mice was significantly increased compared to the healthy controls (mean 37.428 U/gPr versus 28.059 U/gPr, *P* < 0.0001). The activity of CAT in the liver homogenates after treatment of either healthy or tumor bearing mice with SLENU was not significantly higher compared to the healthy controls (mean 32.402 U/gPr and 31.218 U/gPr, *P* > 0.05). One hour after application of BLM, DOX or R the activities of CAT in tumor bearing mice were increased compared to the tumor bearing controls (mean 39.380 U/gPr, *P* > 0.05; 63.667 U/gPr and 55.590, *P* < 0.0001). However, pretreatment with SLENU and following application of BLM, DOX, or R, led to significantly decreased levels of the antioxidant enzyme CAT compared to the groups of tumor bearing mice with BLM, DOX, or R administrated alone (mean 37.199 U/gPr, *P* < 0.001; 30.409 U/gPr and 33.255 U/gPr, *P* < 0.00001). Moreover, CAT activities in all combinations were found to be close to those of the controls. 

### 3.3. Effect of SLENU on Total End Products of NO_2_^−^ and NO_3_^−^ in the Serum

**Figure 5:**
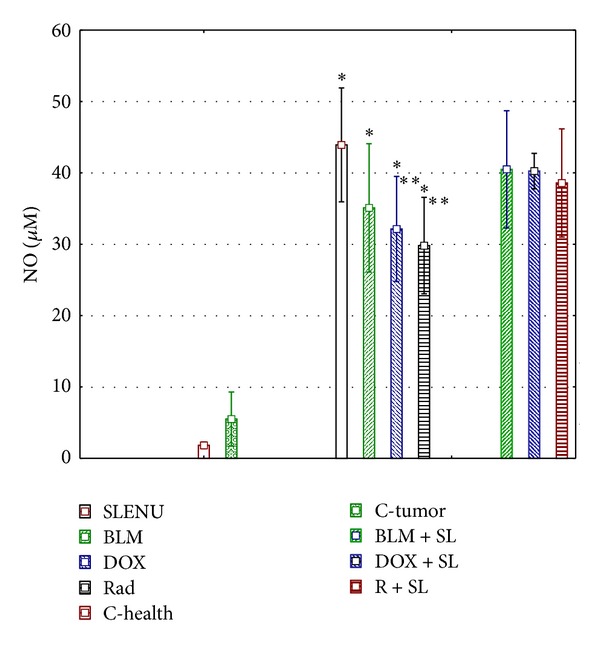
NO^•^ expressed as total end products of NO_2_^−^ and NO_3_^−^. *P < 0.00001 versus controls; **P < 0.0001 versus controls.

Figure 5 shows the levels of NO^•^ expressed as total end products of NO_2_^−^ and NO_3_^−^. The levels of NO^•^ were found to be increased but not significantly in tumor bearing mice compared to healthy controls (mean 5.781 *μ*M versus 1.373 *μ*M, *P* > 0.05). Tumor bearing mice treated with BLM, DOX or exposed to gamma irradiation had remarkably increased levels of NO^•^ compared to the tumor controls (mean 35.252 *μ*M, 33.915 *μ*M, and 30.153 *μ*M, *P* < 0.00001, resp.). It is interesting that mice treated with SLENU had also significantly higher level of NO^•^ than that of tumor controls (mean 44.088 *μ*M, *P* < 0.00001) and also than mice treated with BLM, DOX or exposed to gamma radiation alone, (*P* < 0.0001). Moreover, the levels of NO^•^ for the combinations of BLM, DOX, or gamma irradiation with SLENU were not significantly different from those in mice treated with SLENU alone (mean 40.088 *μ*M, 40.187 *μ*M, and 39.081 *μ*M, *P* > 0.05, resp.). 

## 4. Discussion

The current study was undertaken to evaluate the protective effect of the spin-labeled nitrosourea SLENU against oxidative stress induced in liver of mice treated by antitumor drugs DOX, BLM, or gamma irradiation.

It has already been reported that the stable nitroxyl radical Tempol and other analogues represent a new class of non-thiol-containing radiation protectors that may be useful in elucidating the mechanisms of radiation-induced cellular damage and may have broad applications in protecting against oxidative stress. Further, bearing in mind formerly reported facts by us: (1) an excellent expressed superoxide anion scavenging activity (SSA) of the spin-labeled nitrosourea SLENU and (2) beneficial effects of SLENU on CCNU-induced oxidative stress, we have tried to explain the protective effect of the spin-labeled nitrosourea SLENU on oxidative stress induced by DOX, BLM, and gamma irradiation with possible involvement of free radical mechanisms.

Serum enzyme levels of sGOT and sGPT were measured as primary and specific markers of liver injury. Our results showed increase from three to four times in the levels of sGOT and sGPT for mice treated with either DOX or R alone, compared to the untreated control group, and a slight increase for BLM treated group, compared to the same untreated control group. These findings were in accordance with other authors [9, 10, 21]. After pretreatment with SLENU, the activities of these enzymes were decreased to values similar to those of the controls. 

The levels of lipid peroxidation products (MDA) and the activities of antioxidant enzymes, superoxide dismutase (SOD) and catalase (CAT), in liver homogenates were used as indicatives of oxidative liver injury. To evaluate the oxidative status in liver of treated mice, all measurements were carried out at the 1st hour after the treatment. This time was chosen taking into account our last electron spin resonance (ESR) study by which it was demonstrated that after SLENU i.p. administration, its maximal concentration in lungs, brain, liver, and spleen was reached at 30 min and completely was absent within 90 min in all tissues studied [31]. 

 The results of the present study showed that the levels of MDA and the activities of the enzymes SOD and CAT were found to be significantly changed in tumor bearing mice compared to the healthy controls. This suggests an increased oxidative stress and imbalance in the antioxidant defense in non-treated tumor bearing mice as a consequence of abnormality in antioxidative metabolism due to the cancer process. 

Administration of antineoplastic agents during cancer chemotherapy results in a much greater degree of oxidative stress than that induced by cancer itself. The high level of oxidative stress during chemotherapy may overcome the antioxidant defenses of cancer cells, resulting in lipid peroxides production and interfering with antineoplastic activity [20, 38]. Our results demonstrated that administration of the antitumor drug DOX by i.p. route or total body gamma irradiation caused a much greater degree of oxidative stress than that induced by cancer itself. Immediately 1 hour after treatment with DOX or after total body irradiation, liver homogenates of tumor bearing mice had higher levels of lipid peroxidation products compared to the tumor bearing controls. It was accompanied by disturbance in the antioxidant enzyme defense-increased SOD and CAT activities. After treatment with DOX or after total body irradiation, the oxidative stress and the imbalance of antioxidant enzyme system significantly progress. This disturbance might be due to the augmented generation of toxic reactive oxygen species (ROS) in the liver induced by DOX and irradiation. In addition, these free radicals also mediate oxidation of other cellular molecules and have an important role in the pathogenesis of drug and radiation-induced liver abnormalities. Increased levels of oxidative stress enzymes (SOD, GSH-Px, GR, and CAT) were observed and confirmed in DOX-induced rats [39]. The disturbance in oxidant-antioxidant systems results in tissue injury, which is demonstrated with lipid peroxidation and protein oxidation in the tissue. Several studies have shown that the combination of the inflammatory process, free radical oxidative stress, and lipid peroxidation is frequently associated with liver damage, induced by toxic agents such as DOX [21, 22]. Increased MDA levels and SOD and CAT enzymes activities were found in the liver homogenates of tumor bearing mice after alone treatment with BLM compared to the control mice. Although those increases were not statistically significant, they positively affected by pretreatment of SLENU. This finding additionally confirmed that, at our experimental conditions, BLM acts as a reactive oxygen species- (ROS-) generating drug in liver tissues of mice. Even though the liver is not susceptible to BLM toxicity, apparently some markers of oxidative stress are highly sensitive to this drug. Similar results were previously obtained by other authors, who reported that the hepatic microsomal mixed-function oxidase system is highly sensitive to BLM [4]. The effect of BLM as a ROS-generating antitumor drug was evaluated on antioxidant enzymes and the electron transport system in different cellular fractions of liver in rats [40]. The authors reported that the induced antioxidant enzyme activities in BLM-treated rats may be a response to excessive free radical generation due to BLM metabolism in the animals. 

Another indirect proof for involvement of ROS in drug- and radiation-induced toxicity is the overcome of the oxidative stress by adding typical antioxidants. For example, vitamin E, via its robust free radical scavenging effect, prevents lipid peroxidation and therefore inhibits the hepatotoxic effects of doxorubicin [13]. In order to evaluate the effect of SLENU on BLM, DOX, and R-induced oxidative stress, the tissue levels of MDA and the activities of antioxidant enzymes SOD and CAT were measured after treatment with the combinations of BLM, DOX, or R with SLENU. MDA levels were decreased and antioxidant enzymes SOD and CAT activities were normalized to levels close to the controls. Therefore, with the present study, it was prove a complete overcome of the oxidative stress induced by BLM, DOX, and R when the typical antioxidant SLENU possessing high SSA was added. These results propose that SLENU might be a potential hepatoprotector in doxorubicin, bleomycin, and radiation-induced hepatotoxicity.

Based on this finding, we have hypothesized that if BLM, DOX, and R could generate ^•^O_2_^−^ and ^•^NO *in vivo*, it might contribute to tissue ONOO^−^ and ^•^OH production, and these could be a reason for the oxidative liver injury (increase in MDA level and alteration in SOD and CAT activities) by the following reactions:

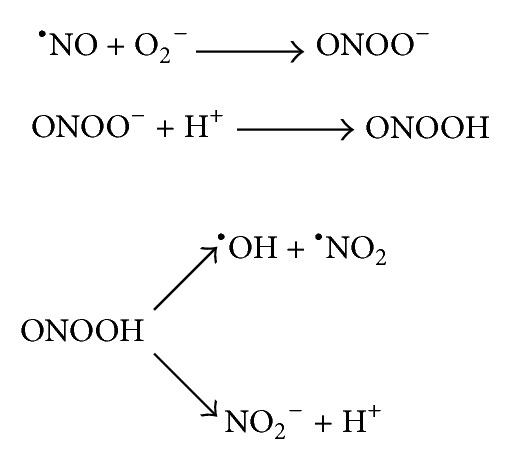
(1)


When mice were pretreated with SLENU, a complete overcome of the oxidative stress in liver homogenates, which is due to DOX, BLM, or R, was observed. A chemopreventive effect of nitroxides such as Tempol was reported by several authors [2, 10, 23]. Authors demonstrated that nitroxides at nontoxic concentrations are effective as *in vitro* and *in vivo* antioxidants, when oxidation is induced by superoxide, hydrogen peroxide, organic hydroperoxides, ionizing radiation, or specific DNA-damaging anticancer agents. The authors explained the protection of oxidative damage by nitroxides through several possible chemical explanations: (1) SOD-mimicking action; (2) oxidation of reduced metals that have potential to generate site-specific ^•^OH radicals; (3) termination of free radical chain reactions induced by alkyl, alkoxyl, alkylperoxyl radical species, and detoxifying drug-derived radicals; and (4) detoxification of hypervalent toxic metal species such as ferryl and cupryl ions.

By EPR studies, we have established that the spin-labeled nitrosourea derivatives, such as SLENU, could successfully scavenge ^•^O_2_^−^ by exhibiting high SSA [28]. We also showed that the mechanism of SSA activity was through redox cycling between nitroxide and its corresponding hydroxylamine moiety, according to the following proposed equations:

(2) N–O•+O•2−+H+→krN–OHN–OH+O•2−+H+→koN–O•+H2O2

where *k*_*r*_ and *k*_*o*_ are second-order rate constants for the reduction of nitroxide and oxidation of hydroxylamine by superoxide, respectively.

The nontoxic effect of the spin-labeled nitrosourea SLENU and its ability to reverse the BLM, DOX, and R-induced oxidative stress in our study have led us to propose the following hypothesis. The nitroso group in the spin-labeled nitrosourea SLENU may lead to the generation of ^•^NO, when SLENU is used alone or jointly with BLM, DOX, and R. However, the nitroxyl-free radical moiety incorporated only in the spin-labeled compounds might successfully compete with the self-generated ^•^NO and that produced by BLM, DOX, and R in the scavenging of ^•^O_2_^−^. This effect could prevent the formation of highly toxic species such as ONOO^−^ and ^•^OH and at the same time could increase the level of ^•^NO. In this regard, our present results are consistent with the notion that the protective effects of SLENU are due to both SSA and its increased release of ^•^NO. 

In our study, serum levels of nitrite (NO_2_^−^) and nitrate (NO_3_^−^) were used to estimate the level of ^•^NO formation, since ^•^NO is highly unstable and has a very short half-life. We observed significantly higher ^•^NO end products in the plasma of mice treated with BLM, DOX, R, and SLENU alone and also in mice treated with the combination of either the drugs or gamma irradiation with SLENU. These results were in agreement with the results reported by other authors. Gurujeyalakshmi et al. reported increase in NO levels as a result from BLM-induced increases in iNOS message and iNOS protein [41]. Irradiated cells produce more NO in response to either IFN-gamma or LPS, and the increase is mediated by induction of TNF-alpha [17, 42]. Several *in vitro* studies have demonstrated the protective effect of ^•^NO in oxidative injury, both in the generalized case and in hepatocytes. Rubbo et al. [43] suggest that ^•^NO may act as a primary antioxidant in biological systems by limiting lipid peroxidative chain propagation. Using a model system, authors demonstrated that ^•^NO is a potent terminator of radical chain propagation and that ^•^NO inhibits peroxynitrite-dependent lipid peroxidation reactions. 

## 5. Conclusions

In view of these facts, we can conclude that the increase in oxidative stress markers and the concomitant change in antioxidant levels indicate the role of oxidative stress in BLM, DOX, and R-induced oxidative liver injuries. Moreover, pretreatment with SLENU shows a protective impact against BLM, DOX, and R-induced oxidative stress and liver injury by scavenging of ^•^O_2_^−^ and increased ^•^NO release. Further studies are, however, needed to clarify the effect of these combinations in antitumor chemotherapy.

## Figures and Tables

**Table 1:** The influence of i.p. administration of SLENU on sGPT and sGOT liver transaminases in BLM, DOX, and R-treated mice.

Compound	sGOT (U/L)	sGPT (U/L)
Controls (health)	60.20 ± 2.60	31.00 ± 0.44
Controls (tumor)	63.27 ± 1.43	36.15 ± 1.74
BLM (60 mg/kg)	78.90 ± 4.10	44.23 ± 0.81
DOX (60 mg/kg)	177.87 ± 2.30*	142.33 ± 1.21*
R	181.80 ± 3.12*	134.56 ± 0.90*
SLENU (100 mg/kg)	62.88 ± 2.04	39.27 ± 1.98
BLM (60 mg/kg) + SLENU (100 mg/kg)	66.50 ± 1.40	41.20 ± 0.62
DOX (60 mg/kg) + SLENU (100 mg/kg)	80.30 ± 1.27^#^	49.89 ± 1.02^#^
R (60 mg/kg) + SLENU (100 mg/kg)	77.45 ± 1.13^#^	37.10 ± 0.77^#^

Data are expressed as mean ± SE; *P < 0.00001 versus tumor controls; ^#^ *P* < 0.0001 versus corresponding DOX or R-treated mice.
